# Risk Stratification of Acute-Onset Chest Pain: SVEAT Score Versus HEART and TIMI Scores

**DOI:** 10.7759/cureus.39590

**Published:** 2023-05-28

**Authors:** Muhammad F Shahid, Asma Malik, Nauman Kashif, Fuad Ahmad Siddiqi, Muhammad Hammad, Hafiz A Saeed

**Affiliations:** 1 Internal Medicine, Pak Emirates Military Hospital (PEMH), Rawalpindi, PAK; 2 Internal Medicine, Combined Military Hospital, Quetta, PAK; 3 Medicine, Combined Military Hospital, Rawalpindi, PAK

**Keywords:** timi score, risk score, risk stratification, chest pain, percutaneous coronary intervention, acute coronary syndrome

## Abstract

Introduction

Acute chest pain is a frequently encountered symptom in the emergency department. Despite the availability of various chest pain risk scores, their effectiveness in identifying low-risk patients suitable for safe and early discharge is inadequate. Moreover, clinical data collected at the initial stage, which has valuable discriminatory ability, is often underutilized. This study aims to assess the effectiveness of the Symptoms, history of Vascular disease, ECG, Age, and Troponin I (SVEAT) score in predicting major adverse cardiovascular events (MACE) in acute-onset chest pain, compared with the pre-existing History, ECG, Age, Risk factors, and Troponin I (HEART) and Thrombolysis In Myocardial Infarction (TIMI) scores.

Methodology

This prospective study utilizing non-probability convenience sampling was conducted in the emergency medicine department of a tertiary care hospital in Rawalpindi, Pakistan, for a period of five months from July 2022 to November 2022. The study included patients aged >45 years who presented primarily with chest pain lasting for at least five minutes but less than 24 hours and with a lack of acute ECG changes indicative of ST-elevation acute coronary syndrome (STE-ACS). Patients who were hemodynamically unstable were excluded. All patients were assessed for the calculation of SVEAT, TIMI, and HEART scores. All patients were followed for a period of 30 days to assess the incidence of MACE.

Results

A total of 60 patients were included. The mean age was 61.5±9.1 years while 31 (51.7%) patients were females. Diabetes was the most prevalent comorbidity (n=32; 53.3%). Regarding MACE, nine (15%) patients developed ACS and underwent percutaneous coronary intervention (PCI). Two patients (3.3%) experienced heart failure. Six (10%) patients also underwent PCI in the absence of ACS while two (3.3%) patients developed sudden cardiac death. Area-under-curve (AUC) values were determined for SVEAT (0.843; 95%CI: 0.74-0.94), TIMI (0.742; 95%CI: 0.62-0.86), and HEART scores (0.840; 95%CI: 0.74-0.94). A cut-off level of 3.5 SVEAT points obtained a sensitivity of 63.2% and specificity of 75.6% in predicting 30-day MACE.

Conclusion

SVEAT score potentially lacks the appropriate sensitivity level to predict a significant number of major adverse cardiovascular events compared to contemporary risk stratification scores. Therefore, the SVEAT criteria need re-evaluation as a screening tool for risk assessment in acute chest pain.

## Introduction

Acute chest pain is characterized as one of the most prevalent global causes of emergency hospitalization. Besides cardiovascular etiology, a multitude of pathological conditions could be manifested in the form of sudden-onset, non-traumatic chest pain. In Pakistan, up to 37% of patients presenting with acute chest pain are likely to be diagnosed with acute coronary syndrome (ACS) [1]. The remaining differential diagnoses can potentially include non-ACS ischemic heart disease (IHD), aortic dissection, pulmonary thromboembolism, pericardial and pleural diseases, and gastroesophageal disorders [2]. A multitude of meticulous scoring systems can be implemented to allow categorization and risk stratification of acute-onset chest pain [3].

The efficacy of a scoring system can be estimated by its capacity to identify patients at a significantly low risk of developing major adverse cardiovascular events (MACE) or mortality during the follow-up period [4]. Classically, the Thrombolysis In Myocardial Infarction (TIMI) and Global Registry of Acute Coronary Events (GRACE) criteria have been implemented for the purpose of risk computation in such cases [5,6]. The overall sensitivity of TIMI in identifying the low-risk cohort approximates 97% whereas GRACE holds a sensitivity of 94% in terms of estimating the all-cause mortality among patients with a clinical suspicion of IHD. Moreover, when using the GRACE criteria, there exists a conspicuous risk of omitting approximately 2.2% of patients who could develop MACE in the follow-up period. Although using the pre-existing History, ECG, Age, Risk factors, and Troponin I (HEART) score can lower this percentage to <1% [7], some authors have identified a higher MACE ratio, which remains undetected through the HEART score [8].

One recently introduced tool is the Symptoms, history of Vascular disease, ECG, Age, and Troponin I (SVEAT) score, which comprises the following parameters: patient’s symptoms and history of vascular disease, ECG findings, individual’s age, and plasma troponin I concentration. In contrast to its counterparts, the initial evidence pinpoints a significantly greater degree of effectiveness for the SVEAT score [9]. Roongsritong et al. have reported that only up to 0.8% of subjects with a low-risk SVEAT score are likely to develop MACE within the 30-day follow-up duration [10]. Therefore, the SVEAT criteria can potentially surpass other contemporary scoring systems.

In this perspective, the current study was designed to assess the competence of the SVEAT score in predicting MACE while conducting risk stratification among individuals with acute chest pain in a tertiary healthcare setting in a developing country.

## Materials and methods

Study design and sampling technique

This project was carried out as a single-centered, prospective, observational study utilizing non-probability convenience sampling. The study was conducted at the emergency medicine department and clinical decision unit of a 1200-bed tertiary care hospital, Pak Emirates Military Hospital (PEMH), in Rawalpindi, Pakistan. The study lasted for a total duration of five months from July 2022 to November 2022. An approximate sample size of 60 was estimated by applying Cochran’s formula as follows:

Sample size = $z^21-\frac{\alpha }{2}[P(1-P)]/d^2$

$z1-\frac{\alpha }{2}$ = Confidence interval (CI) at 95% = 1.96

P = Proportion of patients developing MACE = 19.6% [10]

d= Absolute precision = 10%

Inclusion and exclusion criteria

Patients, aged above 45 years, and presenting primarily with chest pain were included in the study only if they satisfied the following clinical criteria: (i) Duration of painful episode at least five minutes but less than 24 hours, and (ii) Absence of acute ECG changes indicative of ST-elevation ACS (STE-ACS). In addition, hemodynamically unstable patients, patients with noncardiac chest pain due to trauma, those with a prior episode of MACE secondary to IHD, and those unable to provide informed consent were excluded from the study.

Data collection and analysis

At the time of early hospitalization, after informed consent, the SVEAT score of the 60 recruited patients was determined by using the parameters shown in Table 1. ECG recordings and troponin I were tested for all the patients and a thorough history was obtained concurrently. Troponin I value equivalent to 12.8 ng/L was established as the cut-off level for diagnosing ACS [11]. For patients with a strong clinical suspicion of myocardial infarction (MI) but normal cardiac biomarkers, troponin I was serially recorded for up to 12 hours post-admission. Besides SVEAT score, the HEART and TIMI scores were also evaluated at the time of emergency admission and all the essential data required to calculate these scores was obtained at the same time. Treating physicians were responsible for the management of all participants.

**Table 1:** Calculating the SVEAT score in acute-onset chest pain MI: Myocardial Infarction; PCI: Percutaneous Coronary Intervention; SVEAT: Symptoms, history of Vascular disease, ECG, Age, and Troponin I

Parameters	Clinical Presentation	Score
Symptoms	Typical unstable angina	3
	Stable angina	1
	Non-cardiac chest pain	-2
Vascular disease	Recent MI or PCI (< 90 days ago)	2
	Coronary artery bypass grafting >5 years ago	2
	Previous history of coronary events	1
	Previous revascularization procedures	2
ECG changes	New ischemic ST/T changes	3
	ST depression of unknown duration	2
	ST changes with left ventricular hypertrophy, conduction delay, or metabolic abnormalities	1
	Old Q waves	1
	No ST/T changes	0
	Normal ECG in the presence of severe chest pain	-2
Age (years)	>75	2
	50-75	1
	30-49	0
	<30	-1
Troponin I (ng/ml)	0.7 or higher	5
	>0.12 but <0.7	2
	>0.04 but ≤0.12	1
	Normal (≤0.04) with non-specific duration of chest pain	0
	Normal after >4 hours of chest pain	-2

The occurrence of a MACE was characterized as the primary clinical outcome of interest and MACE events were classified as follows: (i) A primary or repeat episode of ACS following the early admission; (ii) Transient ischemic attack (TIA) or ischemic stroke; (iii) Heart failure; (iv) Percutaneous coronary intervention (PCI); (v) Coronary artery bypass graft, and (vi) Sudden cardiac death [12,13]. Following their discharge, all the patients were followed via hospital appointments or via telehealth for a period of 30 days and assessed for the subsequent development of MACE complications.

Before initiating data collection, informed consent was obtained from all the participants while Pak Emirates Military Hospital (PEMH) Ethics Committee issued ethical permission (approval number: A/28/EC/639/2022). Patient-related parameters including age, gender, and underlying medical comorbidities (diabetes mellitus, hypertension, IHD, and TIA/stroke) were recorded into a digitized data collection tool in addition to their SVEAT, HEART, and TIMI scores. For the purpose of statistical analysis, IBM SPSS Statistics for Windows, Version 23.0 (Released 2015; IBM Corp., Armonk, New York, United States) was utilized. Mean ± SD was calculated for the continuous variables included in the study. The receiver operating characteristic (ROC) curves were plotted by using SPSS to estimate the cut-off values for the different scoring criteria. The p-value ≤0.05 was considered significant.

## Results

The mean age of 60 study participants was calculated to be 61.5 ± 9.1 years. Most of the individuals were females (n=31; 51.7%). A total of 24 (40%) patients were chronic smokers (>6 months). Among the medical comorbidities, diabetes mellitus (n=32; 53.3%) was the most prevalent condition followed by hypertension (n=20; 33.3%). Moreover, IHD and stroke/TIA were identified in the medical records of 15 (25%) and five (8.3%) subjects, respectively (Table 2).

**Table 2:** Baseline parameters of participants (n = 60) IHD: Ischemic Heart Disease; TIA: Transient Ischemic Attack

Parameters		Frequency, n (%)
Gender	Male	29 (48.3%)
	Female	31 (51.7%)
Comorbidities	Diabetes mellitus	32 (53.3%)
	Hypertension	20 (33.3%)
	IHD	15 (25%)
	Stroke/TIA	5 (8.3%)
Smoking		24 (40%)

A majority of patients were diagnosed with cardiac pathologies: (i) Unstable angina (n=12; 20%); (ii) Stable angina (n=11; 18.3%); (iii) Non-STE ACS (n=9; 15%), and (iv) Aortic dissection (n=1; 1.7%). Additional provisional diagnoses consisted of gastroesophageal reflux disease/acid peptic disease (n=12; 20%), musculoskeletal disorders (n=10; 16.7%), and pulmonary embolism (n=2; 3.3%). The remaining three (5%) cases were not diagnosed with any specific abnormality. A multitude of MACE complications were identified within the study population. Up to nine (15%) patients developed ACS and were intervened with PCI. Furthermore, two additional patients (3.3%) developed ACS coinciding with acute pulmonary edema/heart failure. A surplus of six (10%) patients also underwent PCI whereas only two (3.3%) patients expired secondary to sudden cardiac death (Table 3).

**Table 3:** MACE events among patients with acute chest pain (Total patients = 60) NSTE-ACS: Non-ST Elevation Acute Coronary Syndrome; GERD: Gastroesophageal Reflux Disease; APD: Acid Peptic Disease; ACS: Acute Coronary Syndrome; PCI: Percutaneous Coronary Intervention; MACE: Major Cardiovascular Events

Provisional Diagnosis	30-day MACE Events (%) (Cumulative Incidence = 19)					Total patients (n = 60)
	No MACE	ACS/PCI	ACS/Heart Failure	PCI	Sudden Cardiac Death	
Unstable Angina	4 (6.7%)	5 (8.3%)	Nil	2 (3.3%)	1 (1.7%)	12 (20%)
Stable Angina	7 (11.7%)	1 (1.7%)	Nil	3 (5%)	Nil	11 (18.3%)
NSTE-ACS	2 (3.3%)	3 (5%)	2 (3.3%)	1 (1.7%)	1 (1.7%)	9 (15%)
Aortic Dissection	1 (1.7%)	Nil	Nil	Nil	Nil	1 (1.7%)
GERD/APD	12 (20%)	Nil	Nil	Nil	Nil	12 (20%)
Musculoskeletal Pain	10 (17%)	Nil	Nil	Nil	Nil	10 (17%)
Pulmonary Embolism	2 (3.3%)	Nil	Nil	Nil	Nil	2 (3.3%)
Non-specific chest pain	3 (5%)	Nil	Nil	Nil	Nil	3 (5%)

**Figure 1:**
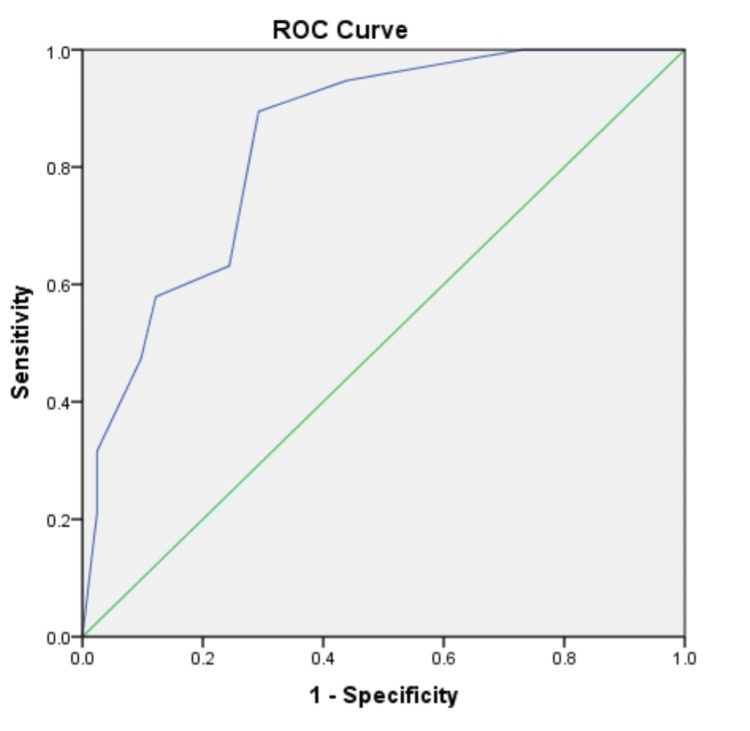
ROC curve for SVEAT score ROC: Receiver Operating Characteristics; SVEAT: Symptoms, history of Vascular disease, ECG, Age, and Troponin I

**Figure 2:**
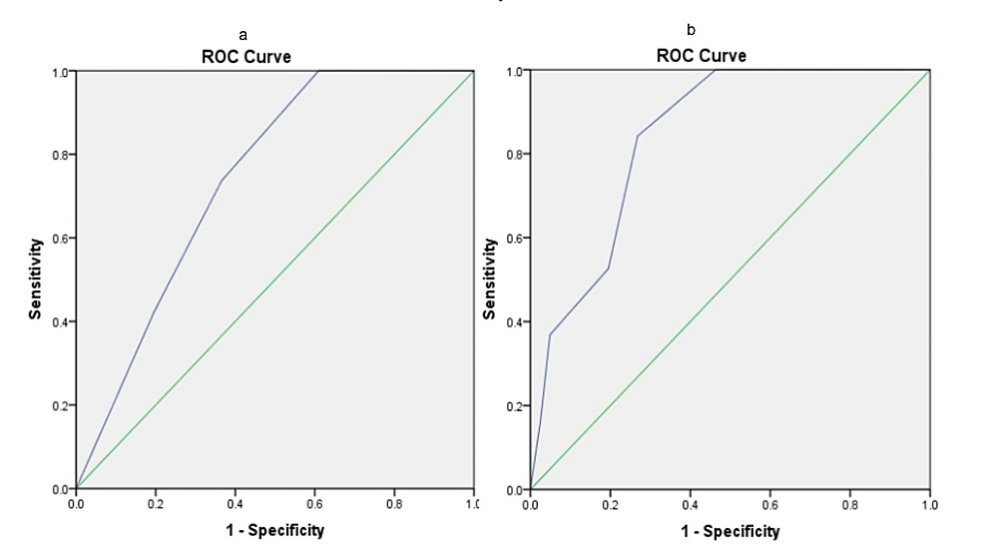
ROC curves for (a) TIMI and (b) HEART scores ROC: Receiver Operating Characteristics; TIMI: Thrombolysis In Myocardial Infarction; HEART: pre-existing History, ECG, Age, Risk factors, and Troponin I

The SVEAT criteria revealed an AUC value of 0.843 (95%CI: 0.74-0.94). To predict the development of MACE, a cut-off point of SVEAT score equivalent to 3.5 obtained a sensitivity of 63.2% and specificity of 75.6% (Figure 1). By using TIMI and HEART scores, AUC was determined to be 0.742 (95%CI: 0.62-0.86) and 0.840 (95%CI: 0.74-0.94), respectively. A TIMI score ≥6.5 predicted MACE with a sensitivity of 73.7% and specificity of 63.4%. For HEART score, a cut-off level of 4.5 achieved a sensitivity and specificity of 84.2% and 73.2%, respectively (Figure 2a-2b). For a score <3.5, a total of seven (36.8%) MACE events were missed by using the SVEAT score. On the contrary, up to five (26.3%) and three (15.8%) MACE events were missed by using the cut-off levels set by the TIMI and HEART criteria.

## Discussion

The study has assessed the applicability of the SVEAT scoring system in the evaluation of acute chest pain at a tertiary care emergency setting in a developing country. When compared to TIMI and HEART scores, the SVEAT criteria possesses a remarkably lower sensitivity yet a higher specificity value. It is, however, noteworthy that at a standard cut-off level of 3.5 points, SVEAT can potentially overlook the 30-day incidence of approximately one-third of MACE events. Currently, only a handful of studies have been conducted to evaluate the predictive role of SVEAT criteria. Hence, these findings hold significance in attempting to replicate data from the study protocol proposed by Roongsritong et al. [10].

Given the fact that chest pain can underlie a plethora of life-threatening cardiovascular morbidities, it is essential to undertake risk stratification at an earlier stage which can help in the differentiation of the high-risk from low-risk patients [14,15]. For this purpose, the SVEAT score provides a reasonable value of sensitivity that is comparable to its predecessors. In the study by Taha et al., which consisted of a total of 321 subjects with chest pain, the SVEAT criteria was effectively capable of isolating up to 73.8% of patients possessing a low risk of developing MACE. Moreover, only a minor proportion (0.8%) of the low-risk study population categorized as having an SVEAT score ≤ 4 experienced MACE events during the initial 30-day follow-up. Contrariwise, up to 1.4% and 1.5% of the patients who were characterized as low-risk by the HEART and TIMI scores, respectively, underwent MACE in the follow-up period [16].

In one study contrasting the overall predictive value of a multitude of cardiovascular scores, the HEART, TIMI, and GRACE criteria lagged in the accurate identification of the incidence of MACE or all-cause mortality among 2%, 3%, and 4% patients, respectively; all of whom were categorized under the “low-risk” category [17]. In a meta-analysis conducted by Ke et al., HEART and TIMI yielded AUC values of 0.8 for the prediction of MACE whereas GRACE had a relatively lower AUC value, i.e., 0.7 [18]. While considering the statistical inadequacy of these standard criteria, the SVEAT scoring system has been applied retrospectively to assess patient admissions secondary to chest pain. Up to 330 eligible records were identified out of whom approximately 3% of patients experienced MACE during their 30-day follow-up. SVEAT score ≤ 4 was designated as a reliable predictor of cardiovascular morbidity when compared to the HEART score [9]. Although this value is comparable to the current findings, further evidence is warranted before the SVEAT score can be efficiently recommended to substitute the established scores for risk stratification of acute-onset chest pain.

The findings from the current study are challenged by a few potential limitations. The study was conducted at a single tertiary care center only and involved a limited size of sample population that was followed for a significantly short-term interval. Moreover, the inclusion of patients with a diagnosis of non-STE ACS as well as non-cardiac conditions could have possibly affected the overall accuracy of study outcomes. It is also noteworthy that the current study lagged in terms of following the six-month follow-up protocol as implemented during the application of the GRACE scoring system.

## Conclusions

In limited studies conducted so far, the SVEAT score emerged as a viable alternative to HEART and TIMI scores for identifying low-risk patients in the risk stratification of acute-onset chest pain. However, our findings indicate that the SVEAT score may not be sufficiently sensitive to accurately predict a significant proportion of MACE when compared to contemporary scores. Consequently, further evaluation is warranted before using the SVEAT score as a routine screening tool for assessing patients with acute-onset chest pain.
